# Safety and Cost Considerations during the Introduction Period of Laparoscopic Radical Hysterectomy

**DOI:** 10.1155/2017/2103763

**Published:** 2017-01-10

**Authors:** A. Anagnostopoulos, S. Mitra, B. Decruze, R. Macdonald, J. Kirwan

**Affiliations:** ^1^Liverpool Women's Hospital NHS Trust, Crown Street, Liverpool L87SS, UK; ^2^Leeds Institute for Biomedical and Clinical Sciences, University of Leeds, Leeds, West Yorkshire LS1 3EX, UK

## Abstract

*Objective*. To compare the safety, efficacy, and direct cost during the introduction of laparoscopic radical hysterectomy within an enhanced recovery pathway.* Methods*. A 1 : 1 single centre retrospective case control study of 36 propensity matched pairs of patients receiving open or laparoscopic surgery for early cervical cancer.* Results*. There were no significant differences in the baseline characteristics of the two cohorts. Open surgery cohort had significantly higher intraoperative blood loss (189 versus 934 mL) and longer postoperative hospital stay (2.3 versus 4.1 days). Although no significant difference in the intraoperative or postoperative complications was found more urinary tract injuries were recorded in the laparoscopic cohort. Laparoscopic surgery had significantly longer duration (206 versus 159 minutes), lower lymph node harvest (12.6 versus 16.9), and slower bladder function recovery. The median direct hospital cost was £4850 for laparoscopic radical hysterectomy and £4400 for open surgery.* Conclusions*. Laparoscopic radical hysterectomy can be safely introduced in an enhanced recovery environment without significant increase in perioperative morbidity. The 10% higher direct hospital cost is not statistically significant and is expected to even out when indirect costs are included.

## 1. Introduction

Radical hysterectomy with pelvic lymphadenectomy is the suggested treatment for cervical cancer FIGO stages 1A2 to 1B1 [1]. In 2010 the National Institute for Clinical Excellence in England (NICE) supported the introduction of laparoscopic radical hysterectomy (LRH) [2]. Since then the laparoscopic approach is being adopted by gynaecological oncology units. High quality evidence for the comparison of open versus the laparoscopic route of radical hysterectomy is lacking [3]. The only reported randomised controlled trial (RCT) included 30 women and revealed superior early postoperative pain outcomes in the laparoscopic arm at the expense of higher major complication rate [4]. An adequately powered RCT would require a sample size up to 1400 patients to detect differences in the safety outcomes between the two techniques [5]. Available evidence is therefore derived from large retrospective case series. The value of such studies in the assessment of implementation of novel surgical techniques has been criticised to obscure results during the learning curve of the technique [6]. The already low incidence of cervical cancer in the developed countries where advanced laparoscopic surgery is commonly available is expected to decrease further following the implementation of HPV vaccination programs. This along with the expanding availability of radiotherapy will eventually decrease the number of radical hysterectomies performed. Furthermore early diagnosis and surgery for small volume disease allow modified radical hysterectomies (type B) for which available comparison data are even less. In the modern practice of higher turnover of surgeons, evidence derived from studies assessing the learning curve of less commonly performed surgical techniques may be of higher clinical relevance.

The implications of enhanced recovery perioperative pathways (ERP) in the clinical outcomes and cost of laparoscopic radical hysterectomy have not been assessed in any reported studies. This study is assessing safety outcomes during the implementation phase of LRH in a tertiary institution. An effort was made to include all short and long-term postoperative complications. Patients presenting with complications to different hospitals were sought and their outcomes were also included. The transparent and honest report of surgical outcomes is essential for clinical governance. Finally the estimation of direct hospital cost during the introduction of the laparoscopic technique and its comparison with this of open surgery is important for the allocation of resources.

## 2. Materials and Methods

Institutional board approval was granted for the study. This single centre study was performed at Liverpool Women's Hospital, a tertiary gynaecological cancer referral centre for Merseyside and Cheshire cancer network which serves a population of 2.3 million. ERP was introduced in January 2011 and LRH in June 2012. Between January 2011 and December 2013 a total of 102 radical hysterectomies were performed for the treatment of cervical cancer and 36 of them were laparoscopic. There were no cases treated with neoadjuvant chemotherapy. After propensity score matching the 36 laparoscopic cases of RH were matched with 36 cases of ARH. The confounders selected for propensity score matching were age, BMI, ASA grade, FIGO stage, and previous abdominal surgery (yes or no). These variables are considered significant for patient selection for surgery and are also associated with outcomes.

In accordance with NICE recommendations LRH was offered to patients selected by the multidisciplinary gynaecological oncology team [2]. The radical hysterectomies performed in both cohorts were mainly B1/2 type and the lymphadenectomies level 1, according to the 2008 Querleu and Morrow classification [7]. Laparoscopic surgery was performed via a 10 mm umbilical optical port and three 5 mm lateral ancillary operative ports. Nerve sparing techniques were not actively pursued.

The direct hospital cost for each procedure was calculated as the sum of three main categories; the cost of surgical time and standard surgical equipment; the length of hospital stay (including the cost of high dependency unit when used); the procedure related perioperative expenses. In this last category we included the cost of consumables, blood products and unscheduled reviews, readmissions, or reoperations within the first 28 days.

Safety outcomes included intraoperative and postoperative complications that were classified according to the Dindo and Clavien proposal [8]. Surgical efficacy outcomes included surgical excision parameters. Perioperative care was standardised for all patients as described at the departmental enhanced recovery pathway. All patients were discharged with an indwelling urinary catheter and readmitted on day 6 for catheter removal. In cases of urinary retention or postvoid urine residuals >100 mL, patients were recatheterised for further 6-7 days. Failure to establish normal micturition after the second week prompted urogynaecology referral. Patient reported outcomes were measured 48 hrs following hospital discharge and collected via standardised telephone interview.

Data were retrieved from electronic patient records and case notes. The sample size was that of convenience. Full propensity score matching was used in order to minimise the mean difference of the distance between the treatment (LRH) and the control group (ARH). Analysis was performed with IBM SPSS statistics 20 (SPSS Inc., Chicago, Illinois) software. Descriptive statistics were produced and normality of distribution was assessed. Comparison of continuous variables was performed with independent sample *t*-test for the normally distributed data and the Mann–Whitney *U* test for the nonnormally distributed ones. In cases of categorical variables Pearson's chi square test was used. The statistical significance of *p* value was set at 5%.

## 3. Results

The ARH cases selected after propensity score full matching presented a mean difference for distance of 0.0073 to the 36 cases of LRH. All 36 patients offered both options of LRH and ARH opted for laparoscopic surgery. There were no significant differences in the clinical and pathological baseline characteristics between the LRH and the ARH control cohort (Table 1).

There were no conversions to open surgery in the LRH group. The intraoperative complication rate for ARH and LRH was 23% and 16.6%, respectively (*p* = 0.5), while the postoperative was 11.1% for both groups (Table 2). There were 3 urinary tract injuries in the LRH group and 1 in ARH group (*p* = 0.3). These included a cystotomy that was identified and treated laparoscopically.

The other 3 were ureteral injuries that presented on the 3rd postoperative week as urogenital fistulas and required reoperation (2 in the LRH and 1 in the ARH cohort). Two vascular injuries involved the internal iliac vein in the ARH group and led to major haemorrhage and HDU admissions. There was 1 obturator nerve injury in the LRH group and 2 genitofemoral nerve injuries in the ARH group resulting in thigh motor deficiency and severe chronic pain, respectively. Blood loss was significantly less in the LRH group both by estimation (mean of 189 versus 934 mL) and by measure of haemoglobin drop (2 versus 4.9 gr/dL). This led to a significantly higher blood transfusion rate in the ARH group (30.5% versus 2.7%, *p* < 0.05). In each group vault dehiscence occurred twice while in the ARH group there was a further case of complete wound dehiscence. Wound, pelvic, or urinary tract infections requiring intravenous antibiotics were more common in the ARH cohort (8.3% versus 2.7%, *p* = 0.3). One episode of deep venous thrombosis was recorded in the LRH cohort. Overall there was no significant difference in complications, according to Dindo and Clavien classification (Table 3).

The number of 28-day unscheduled reviews, readmissions, and reoperations was not significantly different between the 2 cohorts (Table 2).

There was no significant difference in the closest excision or involved margins, neither in the length of vaginal cuff between groups. Lymph node harvest though was significantly higher in the ARH group (Table 4).

As expected the length of surgery was longer for LRH by an average of 45 minutes but the length of stay was shorter by an average of 1.86 days. There was no evidence of recurrent disease in any cohort. Functional recovery of the bladder was significantly delayed in the LRH cohort, with 36.2% of patients requiring bladder catheterisation for more than 1 week compared to 11.1% of the ARH cohort (*p* < 0.05). The degree of severe long-term bladder morbidity was the same with only 1 patient requiring long-term intermittent self-catheterisation in each cohort. Patient reported outcomes showed some delay in bowel function recovery in the LRH cohort while these of mobility, pain control, and oral intake were favourable (Table 5).

The cost analysis showed that the expense of extra operative time and equipment of LRH is almost compensated by the reduced bed occupancy even within an enhanced recovery programme (Table 6).

## 4. Discussion

Safety outcomes during the introduction phase of LRH are comparable with these of standard practice with ARH. Given the option, LRH was preferred patient choice despite the higher urinary tract injury risk quoted during the process of consent. The higher rate of urinary tract injuries during LRH compared to ARH revealed by meta-analysis of large retrospective cohorts [9] was also apparent in this study. Unrecognised urinary tract injuries are a significant cause of morbidity and a low threshold for ureteral stenting may have clinical benefits. We also suggest the addition of a fourth ancillary port so the surgeon can ergonomically complete the dissection of each ureter by standing and operating from the ipsilateral side. Surprisingly the only episode of deep venous thrombosis (DVT) was recorded in the LRH cohort. Despite the lower risk of DVT's associated with laparoscopic surgery [10], the increased lower extremity venous pressure during prolonged Trendelenburg lithotomy positioning along with the CO_2_ pneumoperitoneum can increase this risk [11]. The length of hospital stay for both LRH and ARH in this study was lower than the one quoted in reported studies but the reattendance rate was also high.

The significantly reduced lymph node harvest of LRH has been reported in other studies [2]. Lymph node harvest of both ARH and LRH in this study was lower compared to some reports [2]. This is attributed to the limitation of lymphadenectomies to level 1 [7] and the tailored radicality in small volume tumours. Short-term recovery of bladder and bowel function appeared delayed in the LRH cohort. An increasing number of studies support the fact that laparoscopic [12] and more so robotic [13] surgery facilitate nerve sparing surgical techniques, where branches of the inferior hypogastric plexus are actively sought and preserved. It is suggested that nerve sparing surgery offers better functional postoperative recovery of the pelvic organs [14]. As with all single centre retrospective studies generalisability of results is one of the weaknesses. Prospective multicentre comparative studies of standardised operative techniques using validated patient reported outcome measures during the early and late postoperative period can offer further insight.

The analysis of cohorts with the utilisation of propensity score matching is aiming at reducing the effect of the unavoidable selection bias in these nonrandomised studies. Reported studies in the field have used similar confounders in order to balance their groups [15]. Our operative times and LoS were considerably lower compared to these studies but so was the lymph node harvest. LRH seems to maintain its favourable therapeutic profile over ARH in other reported propensity matched cohorts [16]. A large series of 130 propensity score matched patients reported similar operative times between the two groups, supporting therefore the fact that after the introduction phase of LRH, the cost deriving from the duration of surgery is equalising [17].

Cost analysis in this study refers to the introduction of the laparoscopic technique. It is reasonable to expect that if the indirect cost efficiencies of LRH are considered, the overall cost for LRH is expected to be lower than this of ARH. Introduction of LRH requires upfront investment in equipment and staff training. Financial motives for quality and innovation should be made available to hospitals in order to enable investment in the safe introduction and dissemination of LRH.

## Figures and Tables

**Table 1:** Clinical and pathological baseline patient characteristics.

	LRH (*n* = 36)	ARH (*n* = 36)	*p* value
Age mean	44.6	41.2	0.2
Range (SD)	24–79 (12.2)	24–73 (12.7)	
BMI mean	25.8	26.4	0.5
Range (SD)	19.6–35.8 (3.8)	19.5–34.7 (4.7)	
ASA grade *n* (%)			0.06
1	26 (72%)	18 (50%)	
≥2	10 (28%)	18 (50%)	
FIGO stage *n* (%)			0.2
IA2	1 (2.7%)	0	
IB1	33 (91.8%)	36 (100%)	
IIA1^*∗*^	2 (5.5%)	0	
Histology *n* (%)			0.3
Squamous cell	25 (69%)	20 (55%)	
Adenocarcinoma	11 (31%)	16 (45%)	
Grade *n* (%)			0.3
1	5 (13.8%)	12 (33.3%)	
2	16 (44.4%)	15 (41.6%)	
3	3 (8.5%)	3 (8.5%)	
n/a	12 (33.3)	6 (16.6%)	
Tumor max dimension			0.6
Median	19.5 mm	16 mm	
Range (IQ)	7–37 (30)	9–35 (12)	
Nodal metastasis *n* (%)	4 (11%)	2 (5.5%)	0.3
Previous minor abdominal surgery *n* (%)	7 (19.4%)	13 (26%)	0.1
Follow-up duration (months)	36	36	1

^*∗*^These patients were preoperatively staged as IB1.

**Table 2:** Surgical safety outcomes.

	LRH *n* = 36	ARH *n* = 36	*p* value
*Intraoperative complication*	6 (16.6%)	8 (22.2%)	0.5
Bladder	2 (5.5%)	0	0.1
Ureteric	1 (2.7%)	1 (2.7%)	1
Bowel	1 (2.7%)	0	0.3
Vascular	1 (2.7%)	2 (5.5%)	0.5
Nerve permanent	1 (2.7%)	2 (5.5%)	0.5
Haemorrhage > 1.5 lt *n* (%)	0	3 (8.3%)	0.07
*Postoperative complications*	3 (11.1%)	4 (11.1%)	0.6
Surgical site complications			
Wound/vault dehiscence	2 (5.5%)	3 (11.1%)	0.5
Infection	1 (2.7%)	3 (8.3%)	0.3
VTE	1 (2.7%)	0	0.3
*Others*			
Admissions to HDU	0	2 (5.5%)	0.1
Blood transfusion *n* (%)	1 (2.7%)	11 (30.5%)	<0.05
*28 days *			
A&E reviews	10 (27.7%)	8 (22.2%)	0.5
Readmissions	8 (22.2%)	5 (13.8%)	0.3
Reoperations (minor)	2 (5.5%)	5 (13.8%)	0.2
Reoperations (major)	3 (8.3%)	1 (2.7%)	0.3

VTE: venous thromboembolism, HDU: high dependency unit, and A&E: accidents and emergency.

**Table 3:** Overall complication grade.

Grade	LRH (*n* = 36)	ARH (*n* = 36)	*p* value
I	2 (5.5%)	2 (5.5%)	1
Id^*∗*^	2 (5.5%)	2 (5.5%)	1
II	6 (16.6%)	8 (22.2%)	0.5
IIIb	5 (13.8%)	5 (13.8%)	1
IVa	0	1 (2.7%)	0.3

^*∗*^d: long-term disability.

**Table 4:** Surgical efficacy outcomes.

	LRH *n* = 36	ARH *n* = 36	*p*
Involved excision margins *n* (%)	1 (2.8)	1 (2.8%)	1
Closest excision margin mean in mm	6.8	6.3	0.7
Range (SD)	0.5–14 (4.3)	0.5–20 (5)	
Pelvic lymph node harvest mean (SD)	12.6 (5.3)	16.9 (8)	<0.05
Vaginal vault length mean in mm (SD)	16.7 (9.4)	14.7 (9.8)	0.3
Operation time in minutes mean (SD)	206 (37.7)	159 (48.4)	<0.05
Range	101–268	75–359	
Days of hospital stay mean (SD)	2.31 (0.71)	4.17 (1.34)	<0.05
Range	1–4	2–8	
Bladder function recovery			
TWOC > 1 *n* (%)	13 (36.2%)	4 (11.1%)	<0.05
Urogynaecology review *n* (%)	4 (11.1%)	4 (11.1%)	1
ISC *n* (%)	1 (2.7%)	1 (2.7%)	1
Bowel function recovery			
Constipation *n* (%)	3 (8.3%)	1 (2.7%)	0.3
Adjuvant treatment *n* (%)	5 (13.8%)	1 (2.7%)	0.08
Recurrence	0	0	1

TWOC: trial without catheter and ISC: intermittent self-catheterisation.

**Table 5:** Early postoperative PROM.

	LRH	ARH	*p*
*Mobility*			
Stairs	100%	91.3%	0.1
Out of house	62.5%	63%	0.9
*Analgesia*			
Use of analgesia	63.9%	55.6%	0.7
Effective analgesia	72.2%	58.3%	0.4
*GI*			
Eating & drinking	61.1%	52.8%	0.7
Nausea & vomiting	5.6%	11.1%	0.4
Bowels open	47.2%	63.9%	<0.05
Use of laxatives	69.4%	38.9%	<0.05
*Wound problems*	0	8.3%	0.1

PROM: patient reported outcome and GI: gastrointestinal.

**Table 6:** Direct hospital cost.

Cost of	LRH	ARH	*p*
Standard surgical consumables	715.89	160.12	
Extra perioperative expenses median (IQR)	412.2 (300)	307.5 (371)	0.9
Surgical time mean (SD)	3571.8 (651)	2759 (836)	<0.05
Length of hospital stay median (IQR)	600 (300)	1200 (600)	<0.05
Total median (IQR)	4746.9 (948)	4580.9 (1433)	0.08
